# Haemoglobin and haematocrit: is the threefold conversion valid for assessing anaemia in malaria-endemic settings?

**DOI:** 10.1186/1475-2875-6-67

**Published:** 2007-05-22

**Authors:** Ilona A Carneiro, Chris J Drakeley, Seth Owusu-Agyei, Bruno Mmbando, Daniel Chandramohan

**Affiliations:** 1 Department of Infectious & Tropical Diseases, London School of Hygiene & Tropical Medicine, Keppel Street, London, UK; 2 Joint Malaria Programme, Box 2228, Moshi, Tanzania; 3 Kintampo Health Research Centre, Ghana; 4 National Institute for Medical Research, Tanga Research Centre, Box 5004, Tanga, Tanzania

## Abstract

**Background:**

Anaemic status is determined by haemoglobin using the HemoCue system or haematocrit measurements, and a threefold conversion is commonly used to equate the two measures (haemoglobin = haematocrit/3). The validity of this conversion in malaria endemic settings was assessed.

**Methods:**

Concurrent measures of haemoglobin and centrifuged haematocrit in children aged 6–59 months were compared by modelling the difference between the two measures against their average. A random effects linear regression of the difference of the measures on their average was used to describe the line of best agreement and 95% limits of agreement for these two measures over a range of values after adjusting for statistically significant covariates.

**Results:**

There was a consistent bias between the two measures, with haemoglobin less than haematocrit/3 in 87% (899/1,030) of observations. This difference was non-uniform, decreasing with the average measure, i.e. less difference at higher haemoglobin and haematocrit values. In these studies, use of haematocrit would have underestimated the prevalence of anaemia by misclassifying 10% (89/920) of individuals with haemoglobin < 11 g/dl, 66% (252/380) of individuals with haemoglobin < 8 g/dl and 100% (23/23) of individuals with haemoglobin < 5 g/dl. The mean difference between the measures was greater in males than females, increased with age between 6–59 months, and was greater in the wet than dry season suggesting that the relationship between haemoglobin and haematocrit may be modified by exposure to malaria.

**Conclusion:**

The regression model indicated that the standard threefold conversion from haematocrit to haemoglobin underestimates the prevalence of haemoglobin < 11 g/dl in children under five years of age in malaria endemic settings. This bias was more acute for more severe anaemia defined by haemoglobin < 8 g/dl and haemoglobin < 5 g/dl. This has important implications for the comparability of studies using these different measures. Direct determination of haemoglobin should be the measurement of choice for assessing anaemia outcomes in malaria intervention trials and surveys.

## Background

Anaemia is increasingly being used as an indicator of the impact of malaria control in intervention trials [1-8] and for monitoring and evaluation by the Roll Back Malaria Partnership [9]. Typically anaemia is determined by measuring haemoglobin (Hb) concentration. However, packed cell volume or haematocrit (Hct) has been widely used as an alternative to haemoglobin in malaria studies [1,2,4,10-19]. A standard threefold conversion between the two measures (Hb = Hct/3) is commonly used to define cut-offs for estimating the prevalence of anaemia [20], despite concerns about the accuracy of haematocrit [21-23]. To be able to compare and combine data from multiple studies using different methods of anaemia measurement [24], the comparability of haemoglobin assessed spectrophotometrically using HemoCue and haematocrit was investigated in samples from children aged 6–59 months from two malaria endemic settings.

## Methods

### Sample collection

Data from two studies that have measured haemoglobin and haematocrit were used to assess the reliability of the standard threefold conversion factor. Finger-prick blood samples were collected for determination of malaria and anaemia status from 968 children aged 6–18 months from cross-sectional surveys in the dry and wet seasons of 2000 in Navrongo, Ghana [2] and from 62 children aged 6–59 months from a cross-sectional survey in the dry season of 2001 in Kwemasimba, north-east Tanzania [25]. In both studies, haemoglobin concentration was assessed by haemophotometry (HemoCue, Ängelholm, Sweden), and haematocrit was assessed by centrifugation using standard procedures for Hawksley microhaematocrit tubes and centrifuge (Hawksley & Sons Ltd, Sussex, UK) i.e. 10 minutes at a fixed speed of 11,000 rpm.

### Statistical methods

Bland & Altman [12] argue that two methods designed to measure the same thing will inevitably give a positive linear regression, and the most useful comparison is gained by plotting the difference between the measures against the mean of the two measures. This method was used to compare haemoglobin (g/dl) and haematocrit (%) divided by a factor of three [20] to be able to compare the measurements on approximately the same scale ("grams of haemoglobin per dl"). The difference between the haemoglobin and haematocrit/3 measurements (i.e. Hb - Hct/3) and the mean of the two measurements (i.e. (Hb + Hct/3)/2 – now called average to avoid confusion with mean difference), were calculated for each individual. Linear regression analyses were used to define the relationship between the mean difference and the average of the two measures [26], adjusting for *a priori *covariates of age, sex, season and *Plasmodium falciparum *infection status. A random effects model was used to adjust for potential intra-cluster correlation of observations from the same study. The final regression model was used to define the line of best agreement between haemoglobin and haematocrit, and the 95% limits of agreement were calculated as +/- 1.96 standard deviations (SD) [26]. There was only one average value greater than 14 g of haemoglobin per dl, and this was excluded as it was judged to be an outlier value, and there would be insufficient statistical power to model the relationship above 14 g of haemoglobin per dl.

## Results

**Figure 1:**
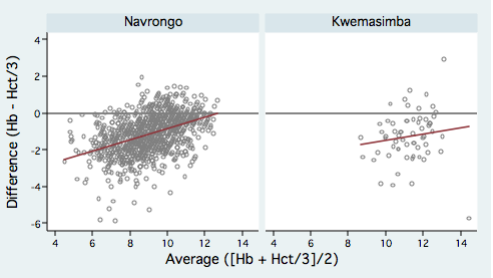
**Scatter-plots of difference against average of haemoglobin and haematocrit/3 for each study**. Scatter-plots of difference against average of haemoglobin and haematocrit/3 for paired measurements from children aged 6–59 months. The line of best fit (red) indicates a trend towards greater differences at lower haemoglobin values. Both axes are in "grams of haemoglobin/dl".

Haemoglobin measurements were lower than haematocrit/3 in both studies, with a linear trend in the relationship so that less negative differences were seen with increasing haemoglobin levels (Figure 1). Logarithmic transformations of the means and differences as suggested by Bland & Altman [27] did not improve this relationship.

The results of the random effects linear regression analysis are presented in Table 1. The difference between haemoglobin and haematocrit showed a statistically significant decrease with the average of the two measures, increased with age, was greater in the wet compared with the dry season and was greater in males than females. The greater difference in individuals with *P. falciparum *infection than in those without in the univariate analyses, no longer remained significant in the multivariate analysis, but was kept in the model as an *a priori *covariate.

**Table 1:** Coefficients from linear regression of difference between haemoglobin and haematocrit/3 (expressed as "g of haemoglobin/dl")

**Covariates**	**Univariate coefficient(95% CI)**	**P-value**	**Multivariate coefficient (95% CI)**	**P-value**
**Average of Hb & Hct/3**				
4.5 "g of Hb/dl"	-3.70 (-4.06, -3.34)^§^	< 0.001	-2.33 (-2.78, -1.89)^§^	< 0.001
Each additional "g of Hb/dl"	+0.28 (+0.24, +0.32)	< 0.001	+0.17 (+0.13, +0.22)	< 0.001
				
**Age:**				
6 – 12 months	-1.07 (-1.14, -0.99)^§^	< 0.001	-2.33 (-2.78, -1.89)^§^	< 0.001
Each additional 1 year of age	-0.26 (-0.36, -0.16)	< 0.001	-0.23 (-0.32, -0.15)	< 0.001
				
***P. falciparum infection*:**				
No	-0.92 (-1.01, -0.84)^§^	< 0.001	-2.33 (-2.78, -1.89)^§^	< 0.001
Yes	-0.58 (-0.71, -0.45)	< 0.001	-0.08 (-0.21, +0.05)	0.23
				
**Season:**				
Dry	-0.57 (-.066, -0.48)^§^	< 0.001	-2.33 (-2.78, -1.89)^§^	< 0.001
Wet	-0.99 (-1.11, -0.87)	< 0.001	-0.66 (-0.80, -0.51)	< 0.001
				
**Sex:**				
Male	-1.28 (-1.37, -1.19)^§^	< 0.001	-2.33 (-2.78, -1.89)^§^	< 0.001
Female	+0.26 (+0.13, +0.39)	< 0.001	+0.21 (+0.09, +0.32)	< 0.001

^§ ^Denotes the constant in the model – i.e. the reference category to which additional coefficients must be added

The final regression model gave the following relationship, which represents the line of best agreement between the two measures:

Diff = - 2.33 + 0.17 × Ave - 0.23 × Age - 0.08 × Pf - 0.66 × Season + 0.21 × Sex

where Diff represents the estimated difference between the two methods, and Ave represents the average of the two methods. Age is in years where 0 actually represents 6–11 months, Pf is the concurrent presence (= 1) or absence (= 0) of *P. falciparum *infection in the child, Season is wet (= 1) or dry (= 0), and Sex is male (= 0) or female (= 1).

A regression of the absolute values of the residuals from this model on the average of the two measures found no relationship (P = 0.252). Therefore, the standard deviation (SD) of the adjusted difference was estimated as the residual SD from the regression model [26], and the 95% limits of agreement were calculated as the regression model ± 1.96 × SD. This is not a confidence interval as it is calculated from the regression model rather than the data, but it provides a reference interval within which 95% of the differences between the two measurements are expected to lie.

In practice, to obtain the estimated difference between the two measurements, Ave can be substituted by any single observed value (i.e. either haemoglobin or haematocrit/3). Equation 1 can be rearranged as

Hb - Hct/3 = -2.33 + 0.17 × Hct/3 - 0.23 × Age - 0.08 × Pf - 0.66 × Season + 0.21 × Sex

to give the predicted haemoglobin value for any given value of haematocrit from:

Hb = (1.17 × Hct/3) -2.33 - 0.23 × Age - 0.08 × Pf - 0.66 × Season + 0.21 × Sex

**Figure 2:**
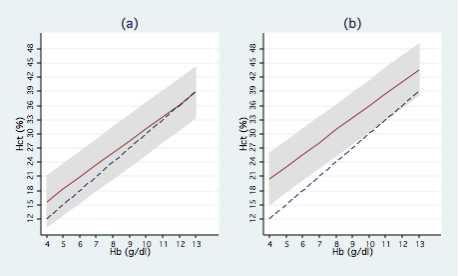
**Line of best agreement between haemoglobin and haematocrit for different scenarios**. The line of best agreement between haemoglobin (g/dl) and haematocrit (%) is given by the solid red line, with the 95% limits of agreement shaded grey. The dashed blue line shows the standard threefold conversion. Graph (a) shows the comparison for females aged 6–11 months with no malaria infection during the dry season, Graph (b) shows the comparison at the other extreme for males aged 4 years with malaria infection during the wet season.

Figure 2 shows the line of best agreement between the haemoglobin and haematocrit measurements for individuals under different scenarios of age, season, sex and *P. falciparum *infection, with the regression-based 95% limits of agreement around these lines. For females aged 6–11 months in the dry season with no *P. falciparum *infection (Figure 2a), the line of best agreement is higher, but not statistically significantly, than the standard threefold conversion, and the two are equivalent above 12 g/dl of haemoglobin. However, for males aged 4 years in the wet season with *P. falciparum *infection (Figure 2b), the line of best agreement is consistently above the standard threefold conversion, and this difference is significant between 4–11 g/dl of haemoglobin. To develop a comparison between haemoglobin and haematocrit for an "average" individual in these settings, average values for age (2 years), sex (0.5), season (0.5) and *P. falciparum *infection (0.5) were substituted into the model. Table 2 shows values from this "average" model and demonstrates how the standard haematocrit cut-offs used to define anaemia (haematocrit < 33%), moderate anaemia (haematocrit < 24%) and severe anaemia (haematocrit < 15%) are likely to underestimate the burden of anaemia compared to haemoglobin measurements using cut-offs of haemoglobin < 11 g/dl, haemoglobin < 8 g/dl and haemoglobin < 5 g/dl respectively. This model suggests that *on average *for children aged 6–59 months in malaria endemic areas, more appropriate cut-offs may be haematocrit < 36% for all anaemia, haematocrit < 28% for moderate anaemia and haematocrit < 21% for severe anaemia. The actual conversion will vary with the covariates discussed above, so the alternative cut-offs presented are indicative of the potential magnitude of the problem of using haematocrit, and are not intended for use as an adjusted conversion table in research or clinical practice.

**Table 2:** Comparison of haematocrit and haemoglobin for an *average *malaria-endemic African population under-5

**Observed Hb (g/dl)**	**Estimated Hct (%) using Hb × 3**	**Actual equivalent Hct (95% limits of agreement)**
4	12	18.1 (12.7, 23.5)
5	15	20.7 (15.2, 26.1)
6	18	23.2 (17.8, 28.6)
7	21	25.8 (20.4, 31.2)
8	24	28.3 (22.9, 33.8)
9	27	30.9 (25.5, 36.3)
10	30	33.5 (28.0, 38.9)
11	33	36.0 (30.6, 41.5)
12	36	38.6 (33.2, 44.0)
13	39	41.2 (35.7, 46.6)

## Discussion

The results showed a consistent bias of haemoglobin measurements to indicate a greater degree of anaemia than haematocrit measurements in the same individuals and populations if the standard threefold conversion is used, as has been reported previously [22]. As this is a secondary analysis of data collected for a different purpose, one potential source of error could be a systematic bias in the haemodilution of the finger-prick sample collection. In both studies a malaria blood smear was made, then the haematocrit sample was taken and finally the sample for haemoglobin. However, differences between haemoglobin and haematocrit measurements have also been described in studies using venous blood [22,23], making this an unlikely explanation for the differences seen here.

Centrifuged haematocrit, as used here, has previously been shown to give falsely elevated results compared with comparative Coulter-derived measurements [28]. However, data from 108 individuals aged 1–4 years from a study in Dielmo, Senegal [29] using Coulter-derived measurement of haematocrit also showed a bias of haematocrit to underestimate anaemia compared with haemoglobin. In that study, haematocrit/3 was consistently 0.72 g/dl of haemoglobin below the haemoglobin measurement but did not vary with any other covariates [24].

The difference between the haemoglobin and centrifuged haematocrit/3 was found to be non-uniform, increasing with average values of these measures, and modified by age, season and sex. These results suggest that the relationship between haemoglobin and centrifuged haematocrit is modified by recent exposure to malaria, as younger age and wet season are both strong correlates of increased malaria exposure.

The error in centrifuged haematocrit measurements can be due to plasma trapping between the packed red cells, and the amount of plasma trapping has been shown to vary according to red cell size (mean corpuscular volume (MCV)), being higher with macrocytes, and increasing with reducing mean corpuscular haemoglobin concentration (MCHC) in hypochromic anaemias [21]. Although plasma trapping was not reported to vary with haemoglobin levels per se, previous results were consistent with a reduced amounts of plasma trapped with increasing haemoglobin levels [21].

Malaria produces normocytic and normochromic anaemia, where the size of the red cells and haemoglobin concentration within the red cells is within the normal range (i.e. MCV = 80–100 fl and MCHC = 320–360 g/l). Red cell destruction during a malaria infection leads to a subsequent increase in erythropoeisis and, thus, increases in the proportion of reticulocytes in individuals recovering from a symptomatic malaria episode [30]. Reticulocytes have a larger MCV and lower MCHC and may act in a similar way to macrocytes and hypochromic cells to increase plasma-trapping following a symptomatic malaria episode. In addition, there are several other conditions that affect red cell size and composition such as micronutrient deficiencies (e.g. folate or iron deficiency) and genetic effects (e.g. alpha thalassaemia, sickle cell disease), and these can be relatively common in malaria endemic settings.

## Conclusion

The conversion between haematocrit and haemoglobin has been shown to vary with age, sex and season of survey, in malaria-endemic settings, and, therefore, there is no simple conversion factor between the two. Thus, while the cause of the differences seen here between haematocrit and haemoglobin measurements is not known, our data argue for the consistent use of haemoglobin rather than haematocrit in the measurement of anaemia in malaria-endemic settings.

## Authors' contributions

IC analysed and interpreted the data, and wrote the first draft of the paper. CD and DC designed and co-ordinated the original field surveys, contributed to data interpretation and to drafting and revising the manuscript. SOA and BM were involved in data collection and critically revised the manuscript. All authors read and approved the final manuscript.

## References

[B1] Shiff C, Checkley W, Winch P, Premji Z, Minjas J, Lubega P (1996). Changes in weight gain and anaemia attributable to malaria in Tanzanian children living under holoendemic conditions. Trans R Soc Trop Med Hyg.

[B2] Chandramohan D, Owusu-Agyei S, Carneiro I, Awine T, Amponsa-Achiano K, Mensah N, Jaffar S, Baiden R, Hodgson A, Binka F, Greenwood B (2005). Cluster randomised trial of intermittent preventive treatment for malaria in infants in area of high, seasonal transmission in Ghana. BMJ.

[B3] Curtis CF, Maxwell CA, Finch RJ, Njunwa KJ (1998). A comparison of use of a pyrethroid either for house spraying or for bednet treatment against malaria vectors. Trop Med Int Health.

[B4] Menendez C, Kahigwa E, Hirt R, Vounatsou P, Aponte JJ, Font F, Acosta CJ, Schellenberg DM, Galindo CM, Kimario J, Urassa H, Brabin B, Smith TA, Kitua AY, Tanner M, Alonso PL (1997). Randomised placebo-controlled trial of iron supplementation and malaria chemoprophylaxis for prevention of severe anaemia and malaria in Tanzanian infants. Lancet.

[B5] Schellenberg D, Menendez C, Kahigwa E, Aponte J, Vidal J, Tanner M, Mshinda H, Alonso P (2001). Intermittent treatment for malaria and anaemia control at time of routine vaccinations in Tanzanian infants: a randomised, placebo-controlled trial. Lancet.

[B6] D'Alessandro U, Olaleye BO, McGuire W, Langerock P, Bennett S, Aikins MK, Thomson MC, Cham MK, Cham BA, Greenwood BM (1995). Mortality and morbidity from malaria in Gambian children after introduction of an impregnated bednet programme [see comments]. Lancet.

[B7] Premji Z, Lubega P, Hamisi Y, McHopa E, Minjas J, Checkley W, Shiff C (1995). Changes in malaria associated morbidity in children using insecticide treated mosquito nets in the Bagamoyo district of coastal Tanzania. Trop Med Parasitol.

[B8] ter Kuile FO, Terlouw DJ, Phillips-Howard PA, Hawley WA, Friedman JF, Kolczak MS, Kariuki SK, Shi YP, Kwena AM, Vulule JM, Nahlen BL (2003). Impact of permethrin-treated bed nets on malaria and all-cause morbidity in young children in an area of intense perennial malaria transmission in western Kenya: cross-sectional survey. Am J Trop Med Hyg.

[B9] Korenromp EL, Armstrong-Schellenberg JRM, Williams BG, Nahlen BL, Snow RW (2004). Impact of malaria control on childhood anemia in Africa – a quantitative review. Trop Med Int Health.

[B10] Akenzua GI, Ihongbe JC, Imasuen IW, Nwobi BC (1985). Anaemia in children: a survey in (Obadan) a rural community in the rain forest zone of Nigeria. J Trop Pediatr.

[B11] Barnish G, Maude GH, Bockarie MJ, Eggelte TA, Greenwood BM (1993). The epidemiology of malaria in southern Sierra Leone. Parassitologia.

[B12] Bjorkman A, Brohult J, Pehrson PO, Willcox M, Rombo L, Hedman P, Kollie E, Alestig K, Hanson A, Bengtsson E (1986). Monthly antimalarial chemotherapy to children in a holoendemic area of Liberia. Ann Trop Med Parasitol.

[B13] Lemnge MM, Msangeni HA, Ronn AM, Salum FM, Jakobsen PH, Mhina JI, Akida JA, Bygbjerg IC (1997). Maloprim(R) malaria prophylaxis in children living in a holoendemic village in north-eastern Tanzania. Trans R Soc Trop Med Hyg.

[B14] Muller O, Becher H, van Zweeden AB, Ye Y, Diallo DA, Konate AT, Gbangou A, Kouyate B, Garenne M (2001). Effect of zinc supplementation on malaria and other causes of morbidity in west African children: randomised double blind placebo controlled trial. BMJ.

[B15] Pietra Y, Procacci PG, Sabatinelli G, Kumlien S, Lamizana L, Rotigliano G (1991). [Impact of utilization of permethrin impregnated curtains on malaria in a rural zone of high transmission in Burkina Faso]. Bull Soc Pathol Exot.

[B16] Reed SC, Wirima JJ, Steketee RW (1994). Risk factors for anemia in young children in rural Malawi. Am J Trop Med Hyg.

[B17] Snow RW, Lindsay SW, Hayes RJ, Greenwood BM (1988). Permethrin-treated bed nets (mosquito nets) prevent malaria in Gambian children. Trans R Soc Trop Med Hyg.

[B18] Snow RW, Rowan KM, Greenwood BM (1987). A trial of permethrin-treated bed nets in the prevention of malaria in Gambian children. Trans R Soc Trop Med Hyg.

[B19] Thomson MC, D'Alessandro U, Bennett S, Connor SJ, Langerock P, Jawara M, Todd J, Greenwood BM (1994). Malaria prevalence is inversely related to vector density in The Gambia, West Africa. Trans R Soc Trop Med Hyg.

[B20] WHO (1968). Nutritional anaemias: Report of a WHO Scientific Group.

[B21] England JM, Walford DM, Waters DA (1972). Re-assessment of the reliability of the haematocrit. Br J Haematol.

[B22] Graitcer PL, Goldsby JB, Nichaman MZ (1981). Hemoglobins and hematocrits: are they equally sensitive in detecting anemias?. Am J Clin Nutr.

[B23] Keen ML (1998). Hemoglobin and hematocrit: an analysis of clinical accuracy. Case study of the anemic patient. Anna J.

[B24] Carneiro I, Roca-Feltrer A, Armstrong Schellenberg J (2006). Estimating the burden of malarial anaemia in children under five years in sub-Saharan Africa. Report from an Agreement for Performance of Work for the Roll Back Malaria Department.

[B25] Drakeley CJ, Carneiro I, Reyburn H, Malima R, Lusingu JP, Cox J, Theander TG, Nkya WM, Lemnge MM, Riley EM (2005). Altitude-dependent and -independent variations in Plasmodium falciparum prevalence in northeastern Tanzania. J Infect Dis.

[B26] Bland JM, Altman DG (1999). Measuring agreement in method comparison studies. Stat Methods Med Res.

[B27] Bland JM, Altman DG (1986). Statistical methods for assessing agreement between two methods of clinical measurement. Lancet.

[B28] Brittin GM, Brecher G, Johnson CA (1969). Evaluation of the Coulter Counter Model S. Am J Clin Pathol.

[B29] Rogier C, Ly AB, Tall A, Cisse B, Trape JF (1999). Plasmodium falciparum clinical malaria in Dielmo, a holoendemic area in Senegal: no influence of acquired immunity on initial symptomatology and severity of malaria attacks. Am J Trop Med Hyg.

[B30] Verhoef H, West CE, Ndeto P, Burema J, Beguin Y, Kok FJ (2001). Serum transferrin receptor concentration indicates increased erythropoiesis in Kenyan children with asymptomatic malaria. Am J Clin Nutr.

